# Nanoparticulate Gels for Cutaneous Administration of Caffeic Acid

**DOI:** 10.3390/nano10050961

**Published:** 2020-05-18

**Authors:** Maddalena Sguizzato, Paolo Mariani, Francesca Ferrara, Markus Drechsler, Supandeep Singh Hallan, Nicolas Huang, Fanny Simelière, Nikul Khunti, Rita Cortesi, Nicola Marchetti, Giuseppe Valacchi, Elisabetta Esposito

**Affiliations:** 1 Department of Chemical and Pharmaceutical Sciences, University of Ferrara, I-44121 Ferrara, Italy; sgzmdl@unife.it (M.S.); hllsnd@unife.it (S.S.H.); mrcncl@unife.it (N.M.); 2 Department of Life and Environmental Sciences, Polytechnic University of Marche, I-60131 Ancona, Italy; 3 Department of Biomedical and Specialist Surgical Sciences, University of Ferrara, I-44121 Ferrara, Italy; frrfnc3@unife.it (F.F.); vlcgpp@unife.it (G.V.); 4 Bavarian Polymer Institute (BPI) Keylab “Electron and Optical Microscopy”, University of Bayreuth, D-95440 Bayreuth, Germany; Markus.Drechsler@uni-bayreuth.de; 5 Institut Galien Paris Sud, CNRS, Université Paris-Saclay, 92296 Châtenay-Malabry, France; nicolas.huang@universite-paris-saclay.fr (N.H.); fanny.simeliere@universite-paris-saclay.fr (F.S.); 6 Diamond Light Source Ltd., Diamond House, Harwell Science and Innovation Campus, Didcot, Oxfordshire OX11 0QG, UK; nikul.khunti@diamond.ac.uk; 7 Plants for Human Health Institute, Animal Science Dept., NC Research Campus, NC State University, Kannapolis, NC 28081, USA

**Keywords:** solid lipid nanoparticles, caffeic acid, poloxamer, small angle X-ray scattering, cigarette smoke

## Abstract

Caffeic acid is a natural antioxidant, largely distributed in plant tissues and food sources, possessing anti-inflammatory, antimicrobial, and anticarcinogenic properties. The object of this investigation was the development of a formulation for caffeic acid cutaneous administration. To this aim, caffeic acid has been loaded in solid lipid nanoparticles by hot homogenization and ultrasonication, obtaining aqueous dispersions with high drug encapsulation efficiency and 200 nm mean dimension, as assessed by photon correlation spectroscopy. With the aim to improve the consistence of the aqueous nanodispersions, different types of polymers have been considered. Particularly, poloxamer 407 and hyaluronic acid gels containing caffeic acid have been produced and characterized by X-ray and rheological analyses. A Franz cell study enabled to select poloxamer 407, being able to better control caffeic acid diffusion. Thus, a nanoparticulate gel has been produced by addition of poloxamer 407 to nanoparticle dispersions. Notably, caffeic acid diffusion from nanoparticulate gel was eight-fold slower with respect to the aqueous solution. In addition, the spreadability of nanoparticulate gel was suitable for cutaneous administration. Finally, the antioxidant effect of caffeic acid loaded in nanoparticulate gel has been demonstrated by ex-vivo evaluation on human skin explants exposed to cigarette smoke, suggesting a protective role exerted by the nanoparticles.

## 1. Introduction

Caffeic acid (CA) is a naturally occurring hydroxycinnamic acid amply present in coffee, fruits, plants, oils, grapes, and tea [1,2]. CA and its derivatives can be considered as strong antioxidants due to different mechanisms, such as radical scavenging activity and inhibition of lipid peroxidation [3,4]. The capability to inhibit or retard oxidation reactions makes CA suitable to counteract inflammatory diseases, aging and some type of tumors [5,6,7]. Indeed CA has photoprotective effect against UVB induced cellular changes and is able to prevent DNA damage induced by free radicals, thus it has been proposed as chemo-preventive agent against cutaneous malignant melanoma [6,7].

Since the skin is the largest and outermost organ of human body, it is exposed to many hazards, such as extreme temperatures, UV radiations, mechanical trauma, as well as biological and chemical agents present in pollution and in cigarette smoke (CS). CS is considered responsible for multiple, highly diverse effects on human health, including tumors, noncancerous lung diseases, atherosclerosis, and disorders of male and female reproductive systems [8,9,10]. In addition, CS can impinge the skin causing mucocutaneous signs, precocious skin aging, chronic dermatoses, and skin cancer [11,12]. Indeed CS components affect cellular redox hemostasis and induce skin inflammation [13]. For instance, some chemicals, such as polycyclic aromatic hydrocarbons, can pass through the epidermis and reach the dermis, leading to systemic effects [14]. The skin can counteract the CS toxic effect by natural mechanisms of defense, acting as antioxidants or oxidant-degrading systems. On the other hand, this homeostatic protection can unsuccessfully increase cutaneous reactive oxygen species (ROS), finally leading to the onset of dermatological diseases [12]. In this respect, the cutaneous administration of antioxidants represents an approach to restore homeostasis, thus preventing ROS-mediated disorders. 

The idea at the basis of the present investigation is the possibility to apply CA on the skin in order to counteract pathologies and disorders induced by CS. Since CA is poorly soluble in water, a nanotechnological formulation has been studied suitable for CA solubilization and able to control CA delivery, prolonging its antioxidant action. Particularly, solid lipid nanoparticles (SLN) have been proposed, being biocompatible non-toxic delivery systems, suitable for inclusion of drugs with different physico-chemical properties in an aqueous physiological environment [15,16,17,18]. Since SLN dispersions don’t possess the adequate viscosity for skin permanence, they need to be thickened, resulting in a final nanoparticulate semi-solid form [19]. Thus, a preformulatory study has been conducted aimed at selecting a polymer for SLN thickening. Namely, poloxamer 407 and hyaluronic acid (HA) have been considered. The former is a copolymer constituted of polyoxyethylene–polyoxypropilene units characterized by thermogelling properties, while the latter is a natural polymer known for wound healing properties and investigated in this study to modulate the formulation strength [20,21,22,23]. The mechanical properties of the nanoparticulate gel have been studied by small angle X-ray scattering (SAXS) and rheological studies, while CA diffusion has been evaluated by a Franz cell. Finally, the CA antioxidant effect has been evaluated ex-vivo on human skin explants exposed to CS.

## 2. Materials and Methods

### 2.1. Materials

The copolymers poly(ethylene glycol)-block-poly(propylene glycol)-block-poly(ethylene glycol) Pluronic F127 (poloxamer 407, p407) (PEO_98_-POP_67_-PEO_98_) and Pluronic F68 (poloxamer 188, p188) (PEO_80_-POP_27_-PEO_80_) were obtained from BASF (Ludwigshafen, Germany). Hyaluronic acid (HA) with molecular weight 300 kDa, was a kind gift of Soliance (Paris, France). Caffeic acid (CA), glyceryl tristearate (tristearin) and nylon membranes (pore size 0.2 µm) were purchased from Sigma-Aldrich (Milano, Italy). Solvents were of high-performance liquid chromatography (HPLC) grade and all other chemicals were of analytical grade.

### 2.2. Preparation of Solid Lipid Nanoparticles (SLN)

Solid lipid nanoparticles (SLN) were produced by a method based on lipid fusion, homogenization and ultrasound treatment. An aqueous solution of p188 (2.5% *w/w*) represented the dispersing phase, while tristearin (5% *w/w*, with respect to the whole weight of the dispersion) represented the disperse phase. Briefly, the p188 aqueous phase (4.75 mL) heated at 80 °C has been added to the molten lipid phase (250 mg) and mixed at 15,000 rpm, at 80 °C for 1 min (IKA T25 digital ultraturrax, IKA-Werke GmbH& Co. KG, Staufen, Germany). The resulting emulsion has been homogenized by ultrasound at 6.75 kHz for 15 min (Microson ultrasonic Cell Disruptor-XL Minisonix, Bioz Inc., San Francisco, CA, USA) and stored at 25 °C. In the case of drug loaded SLN (SLN-CA), CA (0.1% *w/w* with respect to the whole dispersion and 2% *w/w* with respect to the lipid phase) has been rapidly poured into the molten lipid phase and mixed before the emulsification step. 

### 2.3. Photon Correlation Spectroscopy (PCS) Analysis

Dimensional particle analysis was conducted using a Zetasizer Nano S90 (Malvern Instruments, Malvern, UK) supplied with a 5 mW helium neon laser, wavelength output 633 nm. The measurements were repeated thrice at 25 °C at a 90° angle, data were decoded by the “CONTIN” method [24]. 

### 2.4. Cryogenic Transmission Electron Microscopy (Cryo-TEM) Analysis

Solid lipid nanoparticle samples have been firstly vitrified following a method already described [16]. The vitrified species have been moved to a Zeiss EM922 Omega transmission electron microscope (Carl Zeiss Microscopy, GmbH, Munich, Germany) for imaging by a cryoholder (CT3500, Gatan, Pleasanton, CA, USA). Samples were maintained at temperatures below −175 °C during the examination. The specimens have been evaluated with doses of about 1000–2000 e/nm^2^ at 200 kV. The images have been digitally taken by a CCD camera (Ultrascan 1000, Gatan) by an image processing system (GMS 1.9 software, Gatan).

### 2.5. X-ray Scattering

Small angle X-ray scattering (SAXS) experiments have been conducted at TU Graz (Graz University of Technology, Graz, Austria) by using a laboratory SAXS instrument (SAXSpoint 2.0, Anton Paar, Graz, Austria) and at the bioSAXS beamline B21, at Diamond Light Source (Harwell, UK). In the first case, the Cu-Kα radiation (*λ* = 0.154 nm) from a micro-source (point focus) and a Dectris EIGER2 R 500K 2-dimensional area detector (Dectris, Baden-Daettwil, Switzerland) with a pixel size of 75 µm^2^ were used. Since the distance between samples and the detector was 575 mm, the final *Q*-range (being *Q* the modulus of the scattering vector, defined as 4π sin *θ*/*λ*, where 2*θ* is the scattering angle) extended from 0.05 to 4.6 nm^−1^. Samples were placed in a quartz capillary (1 mm diameter, 10 µm wall thickness) with vacuum-tight sealing screw caps at both ends. Measurements were done in vacuum (1 mbar), with an exposure time of 10 min (2 frames): the equilibrium conditions and radiation damage have been carefully monitored. Four different temperatures, namely 20, 30, 37, and again 20 °C, were considered. Equilibrium time of 5 min accounts for constant scattering signal. At Diamond, CA loaded and unloaded SLN were put into PCR tubes in an automated sample changer. The samples were then moved into a temperature-controlled quartz capillary and exposed for 1 s, acquiring 30 frames at 20 °C. Data were collected by a Dectris Eiger 4M (75 × 75 µm pixels) detector with a 3.7 m sample–detector distance and X-ray wavelength *λ* = 0.1 nm. The explored *Q*-range extended from 0.026 to 4.6 nm^−1^. In both cases, 2D data were corrected for background, detector efficiency and sample transmission and were then radially averaged to derive I(*Q*) vs. *Q* curves.

### 2.6. Evaluation of CA Loading into SLN

In order to evaluate CA encapsulation efficiency and loading capacity of CA, SLN samples have been subjected to ultracentrifugation followed by high-performance liquid chromatography (HPLC) [25]. Five hundred microliters aliquot of SLN-CA was poured into a centrifugal filter (Microcon centrifugal filter unit YM-10 membrane, NMWCO 10 kDa, Sigma-Aldrich, St. Louis, MO, USA) and centrifuged (Spectrafuge™ 24D Digital Microcentrifuge, Woodbridge, NJ, USA) at 8000 rpm for 20 min. The lipid phase was then diluted and dissolved with methanol (1:10 *v/v*) for 2 h under stirring. The fraction of aqueous phase was directly evaluated without dissolution. CA content of filtered samples (nylon membranes 0.2 µm pore size) was analyzed by HPLC, as below reported. 

### 2.7. Gel Preparation

P407 gel was prepared by the “cold method” [26] gradually adding an amount of p407 to cold water (5–10 °C) under magnetic stirring, reaching 15 (P15) or 30 (P30) % *w/w* final concentration (Table 1). The vial was sealed and stored in a fridge at 5 °C for 12 h. Two different methods were used to prepare gels containing HA: a “direct method” and a “dilution method”. The "direct method" involved the addition of HA (2% *p/v*) powder to P15 at 4 °C under stirring. The "dilution method" involved a preliminary solubilization of HA (300 kDa) in water (4% *w/v*). The HA solution was then diluted with P30 (1:1, *v/v*) and maintained at 4 °C on an orbital shaker for 24 h. In case of drug containing gels, CA was poured into to the preformed gel and mixed by swirling agitation at a frequency of 30 Hz obtaining a CA content of 1 mg/ml. Nanoparticulate gels were obtained by direct addition of p407 to SLN or to SLN-CA in a 15:85 *w/w* ratio (Table 1). SLN-P and SLN-P-CA were magnetically stirred for 3 h and then kept at 5° C for 12 h up to complete dispersion of poloxamer.

### 2.8. Rheological Analysis

Rheological analysis has been conducted by an AR-G2 controlled-stress rotational rheometer (TA Instruments, New Castle, DE, USA). Particularly the instrument was equipped with an aluminum cone-plate 40 mm diameter, angle 1°, truncation 28 µm and a solvent trap for preventing solvent evaporation throughout the measurements. The viscoelastic properties of the gels (elastic modulus G’ and viscous modulus G’’) have been acquired in oscillation modality. Oscillation frequency was fixed at 1 Hz, while the applied deformations were in the linear regime. Temperature ramps were taken from 5 to 50 °C with a temperature rate 1 °C/min, controlled by a Peltier plate. A 2-min conditioning time at 5 °C was applied before starting the experiments. Measurements were conducted at least thrice for each sample.

### 2.9. Spreadability Studies

After 48 h from gel preparation, the spreadability of P, P-CA, P-HA, and P-HA-CA was evaluated at room temperature (25 °C) [27]. A precise amount of gel, namely 100 mg, was settled on a 3 cm square-Petri dish and then pressed by placing a 50-g mass on a glass dish. The time employed by the gel to fill the total surface of the dish was measured.

The test was carried out three times and the mean values ± standard deviations were obtained by calculating the following equation [27]:

*S* = *m* × *l*/*t*, (1)

where *S* is the spreading capacity of the gel formulation, m is the weight (*g*) carried on the upper dish, *l* is the diameter (cm) of the glass dish, and *t* is the time (s) taken for the gel to fill the total surface. 

### 2.10. In Vitro Diffusion Experiments

Franz-type diffusion cells supplied by LGA (Berkeley, CA, USA) were employed to investigate the CA in vitro diffusion. Briefly, samples of nylon membranes (pore diameter 0.2 µm) (Sigma-Aldrich) were rehydrated by immersion in distilled water at room temperature for 1 h before being fixed in Franz cells with an exposed surface area of 0.78 cm^2^ (1 cm diameter orifice) [25]. The 5 mL of bidistilled water contained in the receptor compartment was maintained under continuous stirring at 500 rpm by a magnetic bar and thermostated at 32 ± 1 °C during all the experiments [27]. In the donor compartment 1 g of each formulation (sol CA, P-CA, P-HA-CA, SLN-CA, and SLN-P-CA) was placed in contact with the membrane surface and the system was sealed to avoid evaporation. Samples of 0.2 mL were withdrawn from the receptor phase at predetermined time intervals comprised between 1 and 8 h, CA content has been analyzed by HPLC. An equivalent volume of receptor phase was replaced after removal of each sample. The mean values ± standard deviations of CA concentrations were calculated after six determinations in independent experiments and plotted as a function of time. The diffusion coefficients, computed from the linear portion of the accumulation curve, were divided by CA concentration (expressed in mg/mL) to obtain normalized fluxes.

### 2.11. HPLC Procedure

HPLC analyses were achieved using a two-plungers alternative pump (Agilent Technologies 1200 series, Santa Clara, CA, USA), equipped with an UV-detector operating at 325 nm and a 7125 Rheodyne injection valve with a 50 μL loop. Samples were injected on a stainless-steel C-18 reverse-phase column (15 × 0.46 cm) packed with 5 μm particles (Platinum C18, Alltech, Apex Scientific, Ronkonkoma, NY, USA). The mobile phase was composed of acetonitrile/water 20:80 *v/v*, pH 2.5, eluted at a flow rate of 0.7 mL/min. Retention time of CA was 4.5 min.

### 2.12. Human Skin Explant Culture

Human skin explants (HSE) were obtained from breast or abdominal tissue samples of healthy adult donors (18–60 years old) undergoing plastic surgery. After subcutaneous fat deletion, 12 mm punch biopsies were rinsed with phosphate buffered saline (PBS) and cultured in Dulbecco’s Modified Eagle Medium (DMEM) containing 100 U/mL penicillin, 100 µg/mL streptomycin, 25 μg amphotericin B (Sigma-Aldrich, Germany), 1% L-glutamine (Sigma-Aldrich, Germany) and 10% FBS at 37 °C in 5% CO_2_ humidified air into 6-well plates [28]. The next day, 20 μL of SLN-P-CA were topically applied for 24 h on HSEs cultured in fresh medium.

### 2.13. Cigarette Smoke Exposure

After 24 h of pre-treatment with the topical application of SLN-P-CA, the HSEs were exposed to cigarette smoke (CS) for 30 min, using 1 research cigarette (12 mg tar, 1.1 mg nicotine). Briefly, as previously described [29], CS was generated by a vacuum pump able to burn the research cigarette. HSEs exposed to filtered air were used as control. After exposure, the culture medium was changed and the explants were incubated at 37 °C in a humidified 5% CO_2_ atmosphere for 24 h. 

### 2.14. Immunohistochemistry

After cutting paraffin-embedded 4 µm sections of skin human explants using a Microtome (Leyca, Buffalo Grove, IL, USA), the sections were deparaffinized in xylene and rehydrated using alcohol solutions with decreasing gradients. For the Antigen retrieval, a heat-based epitope retrieval method was conducted by using 10 mM sodium citrate buffer (AP-9003-500, Thermo Fisher Scientific, Waltham, MA, USA) (pH 6.0). Briefly, the skin sections were submerged in the sodium citrate buffer and placed in a 500 W microwave for 10 min, reaching the temperature of 95 °C. The sections were then cooled for 20 min at 22 °C, washed 2 times in PBS and blocked with 5% bovine serum albumin (BSA) in PBS for 45 min. The primary antibody incubation was performed overnight at 4°C by adding the primary antibodies for 4-HNE (AB5605 Millipore Corp., Burlington, MA, USA) at 1:400 dilution in 2% BSA in PBS. The day after, the sections were washed in PBS for 3 times and then incubated at 22 °C for 1 h with the respective fluorochrome-conjugated secondary antibodies (A11008 Alexa Fluor 488) at 1:500 dilutions in 2% BSA in PBS. After washing the Section 3 times in PBS, a 4′,6-Diamidine-2′-phenylindole dihydrochloride (DAPI) solution (D1306, Invitrogen) 1:20,000 in PBS was used to incubate the skin sections (1 min) and therefore stain the Nuclei. 3 washes in PBS were then performed and the sections were mounted onto glass slides using PermaFluor mounting media (ThermoFisher Scientific) and imaged via epifluorescence on a Leica light microscope equipped at 40× magnification. The fluorescent signal was quantifies using the ImageJ software (ImageJ 1.51j8, National Institutes of Health, Bethesda, MD, USA) [30].

### 2.15. Protein Extraction

Samples for Western blot analysis have been washed with PBS and frozen in liquid nitrogen. The biopsies have been collected in T-PER buffer (Thermo Fisher Scientific, Waltham, MA, USA) added of protease and phosphatase inhibitor cocktails (Sigma-Aldrich, Milano, Italy), in ice-cold conditions. They have been extracted using a bead-based homogenizer at 12,400 rpm at 4 °C for 15 min. Bradford method (BioRad, Hercules, CA, USA) has been employed to measure the protein content [31].

### 2.16. Western Blot Analysis

Equivalent amount of proteins, namely 20 μg for each sample, have been subjected to 12% sodium dodecyl sulfate polyacrylamide electrophoresis gel (SDS-PAGE), electro-transferred onto nitrocellulose membranes, which was then blocked in PBS containing 0.5% Tween 20 and 5% not-fat milk (BioRad). The membranes have been incubated overnight at 4 °C with the primary antibody HO-1 (Abcam, Cambridge, UK) and finally with horseradish peroxidase conjugated secondary antibody for 90 min at 25 °C. The bound antibodies have been revealed in a chemiluminescent reaction (ECL, BioRad). Chemiluminescence has been ascertained on a ChemiDoc imager (BioRad) [32]. The blots have been reprobed with β-actin as the loading control. Images of the bands have been digitized and the densitometry of the bands have been measured using ImageJ software [33].

### 2.17. Statistical Analysis

In the case of nanoparticle characterization experiments, the results are the mean ± SD of 6 determinations, while in the case of ex-vivo tests, data are the mean of 3 analyses obtained in 3 independent experiments. The analysis of variance (ANOVA) was used and a statistical significance was considered at *p*-values < 0.05.

## 3. Results

### 3.1. SLN Production and Characterization

A lipid based formulation has been developed in order to slowly deliver CA on the skin by a not toxic, well tolerated vehicle. SLN dispersions were prepared emulsifying a fused lipid phase constituted of tristearin with an aqueous solution constituted of p188 by hot homogenization, followed by ultrasonication in order to decrease the droplet size. During the emulsification, tristearin droplets were stabilized by p188, afterwards, the droplets solidified into solid nanoparticles under cooling. The final aspect of SLN dispersions was milky and homogeneous. Table 1 reports the composition of unloaded SLN and SLN loaded with CA.

#### 3.1.1. Morphological Analysis

**Figure 1:**
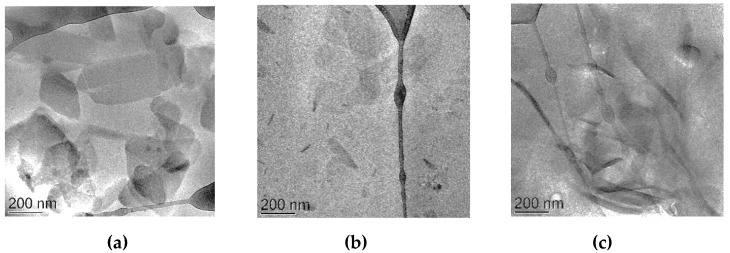
Cryogenic Transmission Electron Microscopy (Cryo-TEM) images of drug loaded solid lipid nanoparticles (SLN-CA) (**a**) and of SLN-P-CA (**b**,**c**).

SLN morphology was studied by cryo-TEM and X-ray diffraction. As shown in Figure 1a, drug loaded SLN (SLN-CA) were characterized by flat irregular elongated particles.

**Figure 2:**
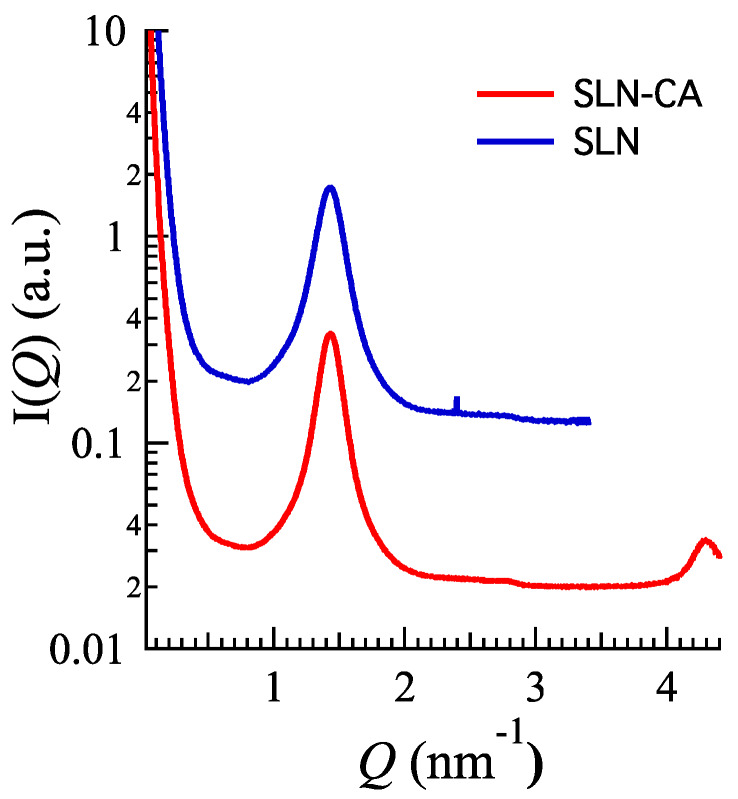
X-ray scattering profile for SLN (blue) and SLN-CA (red) samples. The SLN sample has been measured only up to *Q* = 3.5 nm^−1^, so that the third order is not observable. Experiments were performed at Diamond Light Source (UK).

With regard to inner structure of SLN, X-ray diffraction evidenced a lamellar organization. Figure 2 shows the X-ray profiles corresponding to CA loaded and unloaded SLN: in both cases, Bragg peaks are observed, the position of which scales as 1:2, as expected for a 1D lamellar organization of the lipid matrix (note that the second order has very low intensity; moreover, the SLN-CA sample has been measured in a larger *Q*-range, so that the third order Bragg peak can be appreciated). The unit cell, corresponding to the lamellae repeat distance, is 4.39 nm. Since peak intensity, width and position are independent on the presence or absence of CA, it can be concluded that the drug does not alter the inner structural organization of the SLN.

#### 3.1.2. Dimensional Distribution

SLN and SLN-CA has been analyzed by PCS, in order to have information on their dimensional distribution (Table 2). The mean dimensions of SLN, expressed as equivalent spherical diameters, considering spheres with the same nanoparticle volume, were around 200 nm, with dispersity indexes below 0.3. The presence of CA slightly affected dimensional distribution.

#### 3.1.3. Evaluation of CA Encapsulation Efficiency in SLN

CA was successfully encapsulated within SLN, as determined by analyzing drug content after ultracentrifugation. Indeed CA concentration in SLN-CA was 0.88 mg/mL, namely 88% *w/w* of the drug employed for SLN production was associated to the nanoparticulate lipid phase (1.8% with respect to the lipid phase), while 12% was in the dispersing aqueous phase (Table 2). This result is related to the physico-chemical behavior of CA, being partially soluble in water (log P 1.53). It is interesting to note that SLN enabled to 1.76-fold increase CA solubility with respect to water, where CA is soluble up to 0.5 mg/mL.

### 3.2. Preparation and Characterization of Gels

Since nanoparticulate dispersions don’t possess an adequate viscosity for cutaneous administration, a preformulatory study has been performed in order to select a polymer suitable for SLN thickening. Particularly different polymers have been considered, namely the copolymer PEO_98_-POP_67_-PEO_98_ p407 and HA. P407 is characterized by thermogelling properties, while HA can confer plastic behavior, increasing strength of the final gel [20,21]. Table 3 reports the composition of the gels produced using the polymers alone or in combination. P-HA and P gels were obtained by addition of polymer powder to cold water. Notably CA solubility in P-CA was 1 mg/mL, thus two-fold higher with respect to CA aqueous solution, indeed p407 in water self-aggregates forming micelles that increase drug solubility. In the case of P-HA-CA, a preformulatory study has been conducted to find the best way to combine the two polymers. Direct addition of HA powder to p407 solution resulted in clumps difficult to disperse, thus a method was selected based on 1:1 (*v/v*) dilution of a p407 30% (*w/w*) solution with a HA 4% (*w/v*) solution, finally leading to P-HA-CA, whose composition is indicated in Table 3. 

#### 3.2.1. X-ray Scattering Analysis

**Figure 3:**
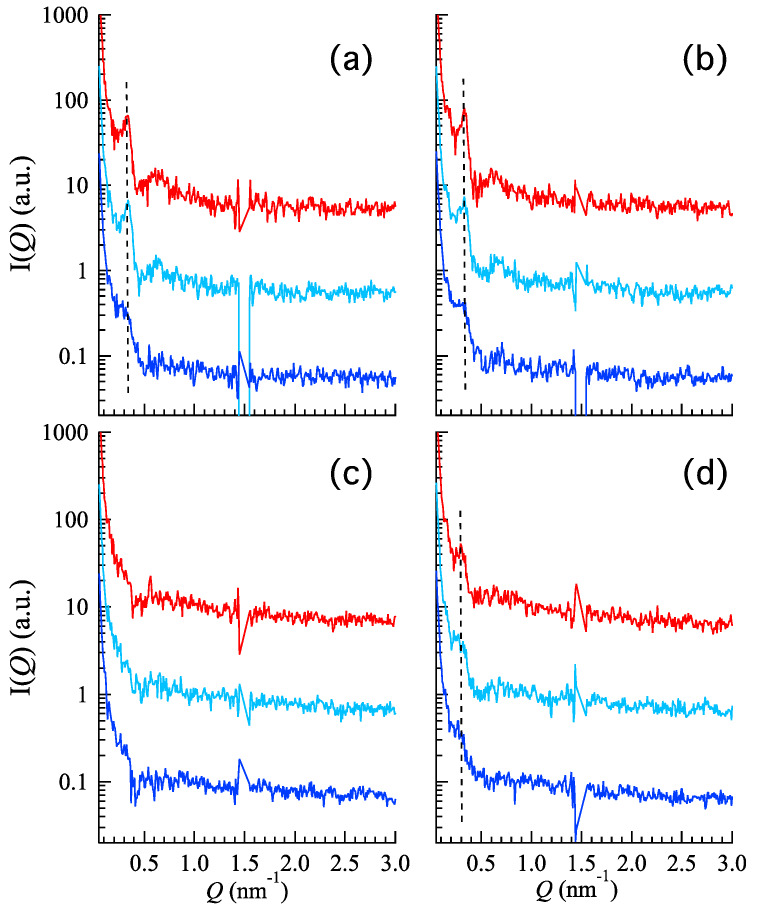
Small angle X-ray scattering (SAXS) diffraction profiles of P (**a**); P-CA (**b**); P-HA (**c**); and P-HA-CA (**d**) at 20 (blue), 30 (light blue), and 37 (red) °C. The dashed line indicates the position of the first correlation peak. Experiments were performed in Graz (AT) laboratory.

In order to investigate the gel supramolecular structure, small-angle X-ray scattering (SAXS) experiments have been conducted on P, P-CA, P-HA, and P-HA-CA at different temperatures (e.g., 20, 30, and 37 °C). Results are reported in Figure 3. Curves are particularly noisy, due to the use of laboratory equipment in combination with a low polymer concentration and a low contrast. In the case of P (Figure 3a), a low-angle, narrow Bragg peak and a broader peak at higher *Q* are clearly detected at all temperatures. The two peaks are in the order 1:√3, according to the occurrence of a pseudo-hexagonal packing order with a lattice constant of ≈22 nm. As expected, the peak intensity of the low-angle peak increases on heating, suggesting that the sample ordering increases as a function of temperature. Indeed, it is well known that by elevating the temperature, the dehydration of the hydrophobic p407 blocks and the hydration of the hydrophilic ones result in spherical micelles formation which successively pack into a 3D lattice. Noticeably, the SAXS profiles (data not shown) confirm the thermoreversible behavior of poloxamer: by decreasing the temperature, the intensity of the low-angle peak decreases, suggesting the disorganization of the micellar network. Similar profiles, together with the same thermal effect, have been found for the P-CA sample (Figure 3b), indicating that the gel supramolecular structure is conserved after the addition of CA. A different behavior is detected in the presence of HA: at one side, P-HA SAXS curves (Figure 3c) are characterized by the absence of peaks at any of the investigated temperatures, while the P-HA-CA sample (Figure 3d) shows the same profile already observed for P and P-CA gels with two differences: (i) at 25 °C no peaks occur and (ii) the peaks observed at 30 and 37 °C correspond to a larger pseudo-hexagonal packing correlation distance (24.2 nm).

As a first conclusion, SAXS data indicate that the p407 based gels have a well-structured organization both in the absence and the presence of CA, with an increasing internal order induced by temperature, as expected considering the temperature responsive nature of the gel [34] and the sol-gel transition below reported. On the contrary, data confirm that HA disorganizes the ordered structure, resulting in the absence of peaks.

In order to better define the gel ordered structure, model fitting of the SAXS data has been performed. According to the accepted structural model, a body-centered cubic lattice of micelle structures with paracrystalline distortion has been considered. The micelles were monodispersed while paracrystal size was infinitely large. Paracrystalline distortions were assumed to be isotropic and characterized by a Gaussian distribution. The model scattering intensity *I*(*Q*)* was calculated as:

*I*(*Q*)* = *k*/*V_p_* × *V*_lattice_ × *P*(*Q*) × *Z*(*Q*), (2)

where *k* is the volume fraction of spheres, *V*_p_ is the volume of the micelles, *V*_lattice_ is a volume correction for the crystal structure, *P*(*Q*) is the form factor of the sphere and *Z*(*Q*) is the paracrystalline structure factor for a body-centered cubic structure [35]. Fitting parameters were the nearest neighbor distance (*D*), the lattice distortion γ, the micelle radius *R* and the electron densities of the micelle and of the solvent.

**Figure 4:**
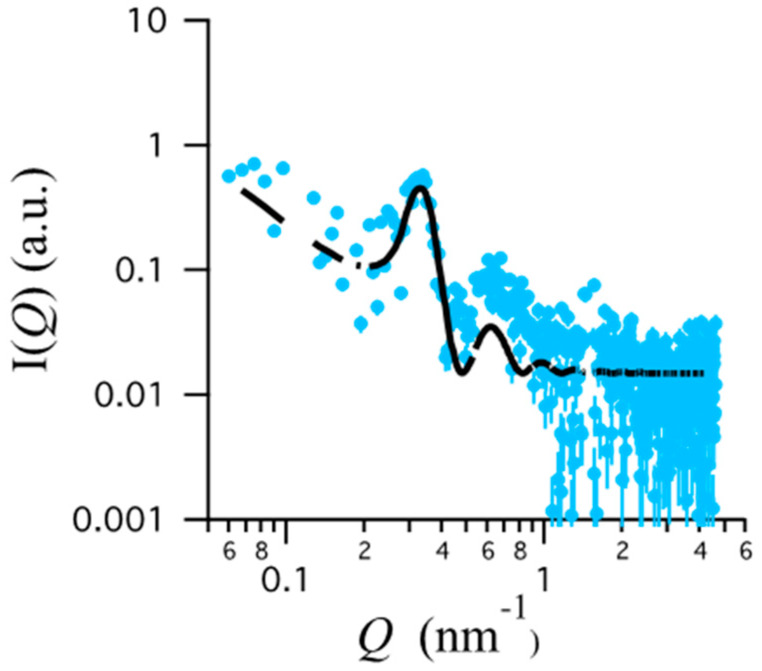
Data fitting for P-CA SAXS curve at 30 °C.

As a kind of example, fitting results are shown in Figure 4, the most relevant fitting parameters are summarized in Table 4.

Even if the SAXS curves are very noisy, the fit is remarkably good. Fitted parameters show that poloxamer molecules, self-assembled into spherical micelles of about 9–12 nm radii, form a gel in which the spherical micelles are packed into a 3D cubic lattice. Thermo-sensitivity of p407 explains the lattice distortion decrease (e.g., the increase of the gel ordering) observed in *P* as a function of temperature. On the other side, the presence of CA appears to induce a further increase of order, while HA increases the lattice distortion. This observation is also confirmed by the absence of Bragg peaks which characterizes the P-HA gel.

#### 3.2.2. Rheological Study

**Figure 5:**
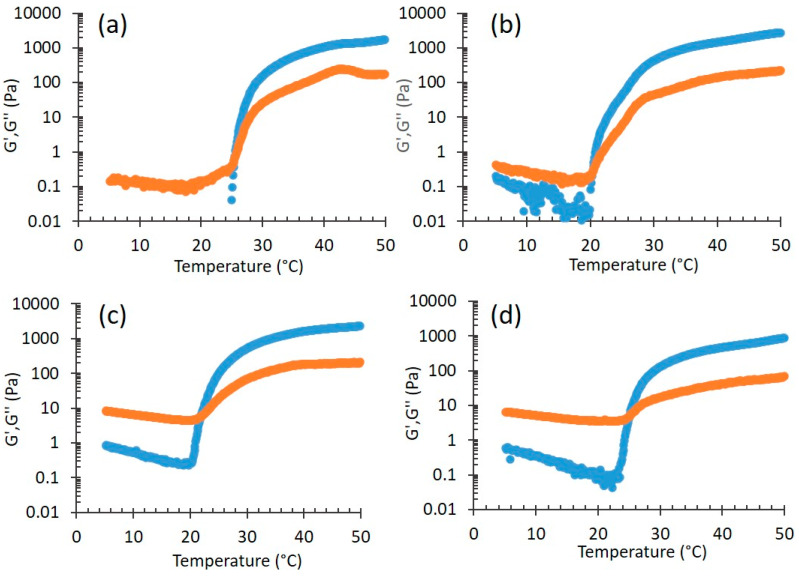
Temperature effect on elastic (G’, blue) and viscous (G”, orange) moduli for P (**a**); P-CA (**b**); P-HA (**c**); and P-HA-CA (**d**).

When p407 gels are used for topical applications, the transition temperature at which the fluid aqueous polymer solution turns to a semi-solid material, *T*_sol-gel_, is one of the most important parameters [36]. Rheology represents a precious way to study the behavior of thermosensitive formulations, since in these systems viscoelastic properties (e.g., elastic G’ and viscous G’’ moduli) depend on their physical state. Therefore, the *T*_sol-gel_ can be easily determined performing rheology experiments as a function of the temperature. Figure 5 reports the G’ and G’’ profiles for P, P-CA, P-HA, and P-HA-CA. In all cases, the thermal behavior is very similar, thus indicating that on heating all formulations became more elastic than viscous, as expected, due to the transition from liquid to structured gel [36]. The maximum elastic modulus, which is related to the strength of the formed gel, is also very similar.

The sol-gel transition temperature was evaluated considering the temperature at which the elastic modulus (G’) and the viscous modulus (G”) are equal [37]. The resulting *T*_sol-gel_ values are shown in Table 5. A few points should be evidenced: first, the comparison between P and P-CA profiles (in the case of P-CA the gel formed at a lower temperature with respect to *P*, with a *T*_sol-gel_ difference of 4.2 °C) suggests that the presence of CA increased the system order, as indicated by SAXS results. Second, P-HA-CA displays a *T*_sol-gel_ value higher than P-HA (the difference is 2.8 °C), indicating a less ordered structure, in agreement with X-ray scattering findings. Indeed SAXS and rheology are complementary, because the former characterizes the supramolecular structure of samples, while the latter defines the macroscopic behavior [38].

#### 3.2.3. Gel Spreadability Study

Spreadability represents a technological parameter affecting the extrusion capacity of semisolid forms from the package, their capability to cover skin area, the patient compliance and definitely the therapeutic efficacy of drugs [27]. At this regard, to gain information on their cutaneous administration, the gel spreadability values have been evaluated (Table 5). All the gels possess a suitable spreadability for cutaneous administration. Nonetheless, P and P-CA were less spreadable with respect to HA containing gels, suggesting that the presence of HA reduced gel consistence. CA did not affect spreadability of both gels. 

#### 3.2.4. In Vitro CA Diffusion Kinetics from Gels

**Figure 6:**
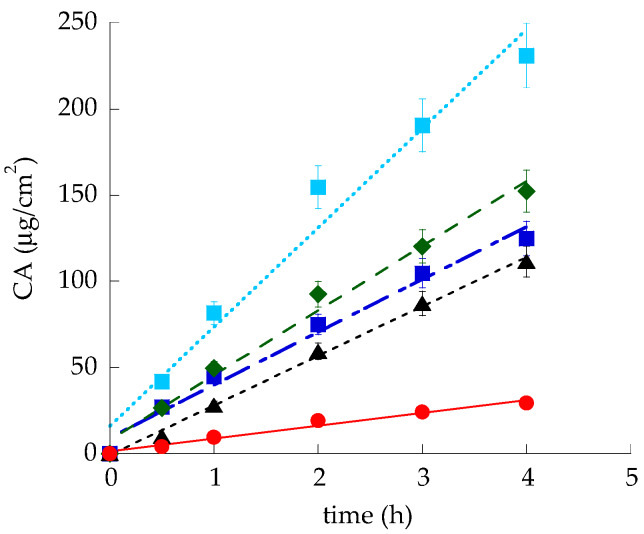
CA diffusion kinetics from Sol-CA (light blue), P-HA-CA (green), P-CA (blue), SLN-CA (black), and SLN-P-CA (red), as determined by Franz cell. Data are the mean of 6 independent experiments.

In order to verify the suitability of gels designed for topical administration and to select the polymer to employ for SLN thickening, the in vitro CA diffusion was studied by Franz cell associated to nylon membranes. Particularly P-CA and P-HA-CA have been considered and compared to the plain CA aqueous solution (CA-sol), as reported in Figure 6.

Diffusion profiles fitted well with zero-order kinetics. It should be considered that in the case of sol-CA, CA concentration was 0.5 mg/ml, thus the diffusion coefficient (D), obtained dividing flux by CA concentration within the formulation, was double with respect to the flux (Table 6). CA fluxes from both gels were slower with respect to sol-CA, particularly in the case of P-CA. Indeed the D of CA from P-HA-CA was 3.13-fold lower than sol-CA, while in the case of P-CA, D was 3.73-fold lower.

On the basis of the obtained results, since P-CA (i) displayed the lowest *T*_sol-gel_ and spreadability values and (ii) was able to better control CA diffusion, p407 was selected for SLN thickening.

### 3.3. Preparation and Characterization of Nanoparticulate Gels

SLN thickening has been achieved by direct addition of p407 to nanoparticle dispersions. The compositions of the obtained nanoparticulate gels are reported in Table 1. The addition of p407 increased consistency but did not affect the macroscopic aspect of nanoparticulate forms, being milky and homogeneous. Cryo-TEM images, reported in Figure 1b,c, show the presence of elongated irregular flat particles that appear thinner and electron dense when observed on side view. Mean dimensions of SLN-P and SLN-P-CA were almost unvaried, being 10 or 20 µm-higher with respect to plain SLN and SLN-CA, as measured by PCS. As expected, encapsulation efficiency values were very slightly affected by p407 addition (Table 2), indeed the polymer has been directly added into pre-formed SLN-CA, where the drug was previously loaded. Moreover the presence of p407 in the aqueous dispersing phase of SLN-CA resulted in the formation of micelles, probably embodying an amount of CA present in the aqueous phase.

#### 3.3.1. X-Ray Scattering Analysis

**Figure 7:**
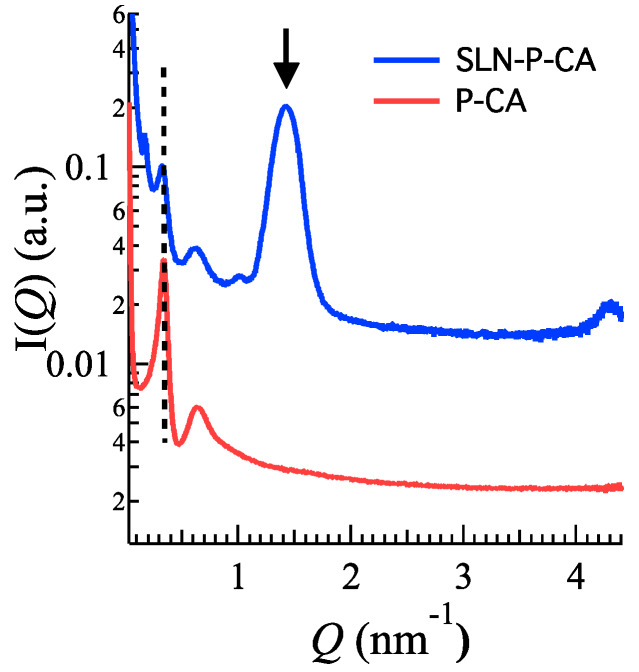
X-ray scattering profiles for P-CA (red) and SLN-P-CA (light blue) samples measured at 37 °C. The dashed line indicates the position of the gel first correlation peak; the arrow points the position of the first Bragg peak related to the inner organization of the SLN. Experiments were performed at Diamond Light Source (UK).

X-ray diffraction experiments on the nanoparticulate gels confirmed that both the nanoparticle inner structure and the whole gel structure were conserved. Indeed, Figure 7 clearly shows that the scattering profile of SLN-P-CA combines the characteristics of the SLN-CA profile (Figure 2) plus the ones of the profile observed for P-CA (Figure 3b and Figure 7, the second referred to a P-CA sample measured at the same beam-line). Notably, peak positions were very similar: the spacing of the Bragg peaks assigned to SLN scales as 1:2:3 (the second order peak has a very low intensity also in this case, see Figure 2), confirming the 1D lamellar organization of the lipid matrix. As a further confirmation of the absence of effects on the structural properties of nanoparticles, the lamellae repeat distance resulted 4.38 nm (4.39 nm in SLN-CA). The position of the low angle peaks corresponding to the gel structural organization (the first narrow and the second rather large, according to the low-degree of order previously discussed) were in the order 1:√3, indicating the preservation of the paracrystalline micellar packing with a lattice constant of ≈21.4 nm [35]. Accordingly, in the case of P-CA the lattice constant was confirmed to be ≈22.0 nm.

#### 3.3.2. Rheological and Spreadability Studies

**Figure 8:**
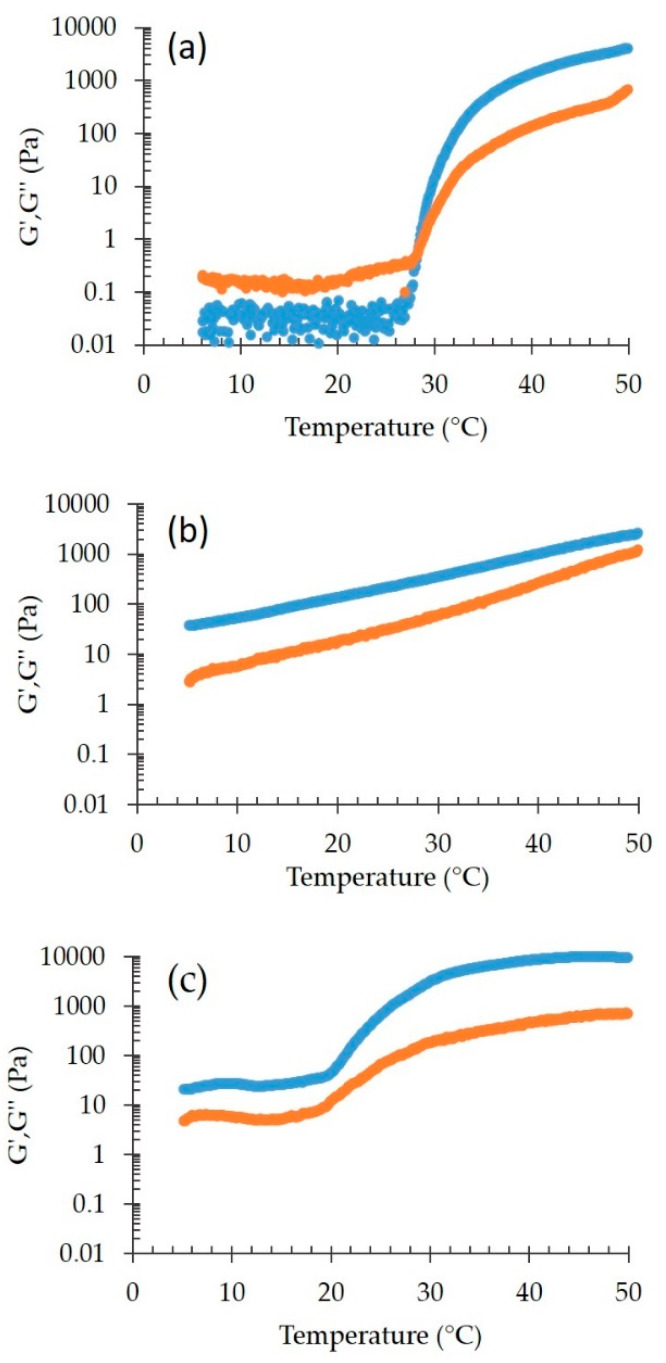
Temperature effect on elastic (G’, blue) and viscous (G”, orange) moduli for P-P188-CA (**a**), SLN-CA (**b**), and SLN-P-CA (**c**).

The effect of p407 addition has been evaluated on rheological behavior and spreadability of nanoparticulate gel. Particularly the thermosensitive behavior of SLN-P-CA has been compared to plain SLN-CA and to a mixture of p407/p188 containing CA (P-P188-CA) at the same concentration used for SLN production. Figure 8 shows the effect of the temperature on G’ and G” moduli. 

In the case of P-P188-CA (Figure 8a), the G’/G’’ profile was similar to P-CA (Figure 5a), eventhough the presence of p188 delayed significantly the gelation temperature, up to 7 °C with respect to P-CA. This behavior could be attributed to the formation of mixed p188/p407 micelles that organized themselves in a different way with respect to the compact paracrystalline structure found in P-CA gel [20,35]. Conversely, the rheological profile of SLN-CA (Figure 8b) was characterized by the absence of crossover, with G’ higher than G’’, indicating a more elastic than viscous behavior for the whole temperature range. The differences between P-P188-CA and SLN-CA profiles suggest that p407 governed the copolymer mixture profile, while p188 did not affect SLN-CA rheological behavior. In the case of SLN-P-CA the addition of p407 to SLN did not modify this trend, but a sudden increase of the moduli could be noticed at ≈20 °C. Thus both SLN and p407 had a crucial effect on the rheological behavior of the gel. The differences between SLN-P-CA and P-P188-CA profiles should be ascribed to the presence of lipid nanoparticles that disorganized the micellar system formed in the case of the mixture of copolymers.

Even though it was not possible to precisely define the *T*_sol-gel_ of SLN-P-CA, the system was characterized by a soft gel consistency, as confirmed by the spreadability values reported in Table 5. It should be underline that the spreadability of SLN and SLN-CA was not measurable because of their liquid state. On the other hand, the addition of p407 to SLN and SLN-CA enabled to obtain systems whose spreadability values were closed to those of P and P-CA.

#### 3.3.3. In Vitro CA Diffusion Kinetics

CA diffusion from SLN-P-CA has been investigated by Franz cell. As shown in Figure 6, the diffusion profile of CA from the nanoparticulate gel was the slowest, indeed D was eight-fold lower than CA-sol and four-fold lower with respect to P-CA. This result suggests that the association between nanoparticles and the entanglement of p407 copolymer chains enabled to slow down CA diffusion.

### 3.4. Ex-Vivo Evaluation of SLN-P-CA in Protecting Human Skin against Oxidative Damage

#### 3.4.1. Immunofluorescence Staining

The skin represents the main barrier of our body against environmental insults and several studies have investigated the effects of outdoor stressors on cutaneous tissues [39]. For instance, CS, a well-known environmental stressor, has been shown to be able to interact with lipids present within the stratum corneum of the skin, leading to the generation of free radical species and lipid peroxidation products, such as 4-hydroxynonenal (4-HNE) [40,41]. Therefore, in order to study the effect of CA delivered by SLN-P in protecting skin against oxidative insults, an immunohistochemical analysis for 4-HNE protein adducts levels has been performed on HSE with or without SLN-P-CA exposed to CS for 30 min, as reported in the method section, at different time points (0 and 6 h).

**Figure 9:**
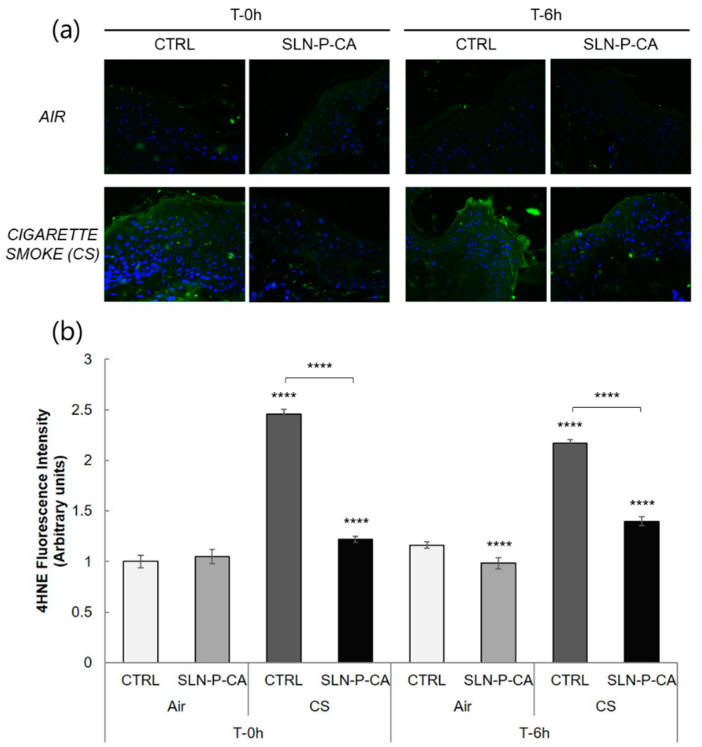
Representative images of immunohistochemical analysis for 4-HNE (green) and DAPI (blue) in ex vivo human skin biopsies at 40× magnification. Immunofluorescence on human skin explants (HSE) was determined instantly and 6 h after exposure of CS for 30 min (panel **a**). Quantification of immunofluorescence signal by using ImageJ software (panel **b**). Data are the average of three experiments. **** *p* ≤ 0.0001 vs control (CTRL) Air at the same timepoint.

As shown in Figure 9, the HSE exposed to CS display a significant increase in 4-HNE protein adduct levels compared to the not exposed skin explants (Air), both at 0 and 6 h. Moreover, the pre-treatment with SLN-P-CA was able to counteract the oxidative damage induced by CS exposure, as indicated by the decrease in 4-HNE fluorescence intensity level in the SLN-P-CA treated HSE compared to untreated ones. 

#### 3.4.2. Western Blot Analysis

**Figure 10:**
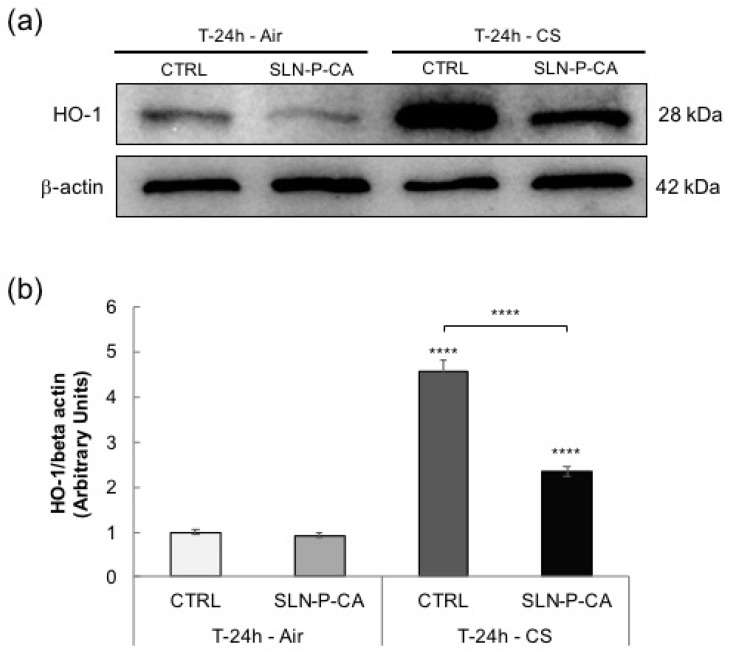
Expression of heme-oxygenase (HO-1) protein levels on HSE exposed to air or CS for 30 min and pre-treated with SLN-P-CA. Samples were collected after 24 h from exposure. Western blot analysis of HO-1 protein expression is representative of three experiments and β-actin was considered as control (panel **a**). Quantification of HO-1 expression bands as a ratio of β-actin (panel **b**). **** *p* ≤ 0.0001 with respect to control (CTRL) Air.

In order to investigate the effect of CA involved in the cutaneous antioxidant response, Heme oxygenase-1 (HO-1) protein levels (stress-response enzyme [42,43]) were also evaluated in the different samples. HO-1 has been studied by Western blot analysis 24 h after CS exposure, quantified by densitometry and normalized by the beta-actin level for each sample, as depicted in Figure 10. Our results demonstrate that the pre-treatment with SLN-P-CA was able to counteract the significant increase in HO-1 protein levels induced by CS after 24 h, confirming the result obtained with immunohistochemical analysis and underlying the ability of the CA containing nanoparticulate gel in preventing skin oxidative stress damage. 

## 4. Discussion

The results obtained in the present study corroborates the idea of using a nanoparticulate gel for applying CA on the skin, preventing and controlling oxidative stresses such as exposure to CS and pollution. The inclusion of CA in SLN has been previously proposed by Fathy and colleagues to obtain a formulation suitable for CA colon delivery [44]. In order to control CA release, the authors relied on a strategy for coating SLN by biopolymers. In the present investigation, SLN-CA were simply spiked with p407, resulting both in thickening and in control of CA diffusion.

The choice of the thickener was made upon evaluation of the biodegradable p407 and HA polymers. Despite other authors have found that the association of p407 and HA led to an improvement of the viscoelastic properties of p407 gel [23], we found that the structure of P-HA-CA was less ordered with respect to P-CA, as indicated by the complementary SAXS and rheological analyses [38]. It is noteworthy that the results obtained by these last methodologies agree well, indicating that the presence of CA increased the system order in P-CA, whilst HA disorganized the structure. Therefore, the addition of HA substantially resulted in a gel strength reduction, as previously found by Wei and colleagues [34]. Indeed P-HA-CA was characterized by *T*_sol-gel_ and spreadability values higher with respect to P-CA, consequently CA diffusion from P-HA-CA was faster.

The addition of p407 to SLN-CA preserved nanoparticle morphology, as demonstrated by cryo-TEM and SAXS, and scarcely affected dimensions, suggesting in SLN-P-CA the presence of nanoparticles embedded within the p407 3D paracrystalline cubic structure.

Moreover, p407 presence allowed to slightly decrease SLN-P-CA spreadability with respect to P-CA. Notably, the formulation should be enough spreadable to be squeezed from the package on one hand and to remain in the application site on the other. The final supramolecular organization of SLN-P-CA enabled to slow down CA diffusion more efficaciously with respect to the other formulations.

Remarkably, SLN-P-CA were able to long control CA antioxidant effect, as corroborated by ex-vivo evaluation on HSE, suggesting that the effect of CA is a long lasting effect. It is possible indeed that CA does not only quench the free radicals present in CS, but stimulates the cellular antioxidant response by activating the NRF2 pathway, as already observed by other authors [45]. Further studies will be performed in order to better understand the level of penetration of this formulation, based on time and dose in different in vivo models. This would allow to understand whether the topical application could have even a protective effect not only in the upper epidermis but even at the lower cutaneous layers, such as basal epidermis and dermis.

## Figures and Tables

**Table 1:** Composition of the indicated formulations, expressed as percentage by weight.

Nanoparticulate System	Tristearin	p188 ^1^	Water	CA ^2^
SLN	5	2.38	92.62	-
SLN-CA	5	2.38	92.52	0.1

^1^: poloxamer 188; ^2^: caffeic acid.

**Table 2:** Size distribution and encapsulation parameters of the indicated nanoparticulate systems.

Nanoparticulate System	Z-Average (nm) ± s.d. ^1^	Dispersity Index ± s.d. ^1^	Encapsulation Efficiency (%) ^2^	Loading Capacity (%) ^3^
SLN	216 ± 12	0.28 ± 0.02	-	-
SLN-CA SLN-P SLN-P-CA	201 ± 11 225 ± 10 230 ± 14	0.29 ± 0.03 0.23 ± 0.04 0.26 ± 0.03	88.2 ± 8.3 - 87.8 ± 5.2	1.8 ± 0.03 - 1.7 ± 0.05

^1^ as determined by PCS; ^2^ Percentage (*w/w*) of CA within the SLN disperse phase, with respect to the total amount of CA employed. ^3^ Percentage (*w/w*) of CA within the SN disperse phase, as compared to the amount of lipid used. Data represent the mean ± s.d. of 6 independent experiments.

**Table 3:** Composition (% *w/w*) of the indicated formulations.

Gel System	Tristearin	p188 ^1^	p407 ^2^ HA	HA ^3^ HA	Water HA	CA ^4^ HA
P P-HA P-CA P-HA-CA SLN-P SLN-P-CA P-P188-CA	- - - - 4.25 4.25 -	- - - - 2.02 2.02 2.02	15.0 15.0 15.0 15.0 15.0 15.0 15.0	- 2.0 - 2.0 - - -	85.0 83.0 84.9 82.9 78.52 78.42 82.88	- - 0.1 0.1 - 0.1 0.1

^1^: poloxamer 188; ^2^: poloxamer 407; ^3^: hyaluronic acid; ^4^: caffeic acid.

**Table 4:** Fitting parameters of SAXS data of gels.

Fitting Parameter	P (20 °C)	P (37 °C)	P-CA (37 °C)	P-HA-CA (37 °C)
*D* (nm) lattice distortion micelle radius (nm)	24.8 0.18 7.9	24.7 0.14 9.4	25.0 0.11 10.5	26.3 0.12 12.1
reduced chi-squared	10.5	6.4	5.5	5.3

Errors are in the order of 5% for *D* and micellar radius and of 10% for the lattice distortion.

**Table 5:** *T*_sol-gel_ and spreadability parameters of the indicated gels.

Gel	*T*_sol-gel_	Spreadability (g × cm/s)
P P-CA P-HA P-HA-CA P-P188-CA SLN-P	25.1 ± 3.1 20.9 ± 2.1 22.0 ± 2.2 24.8 ± 5.2 28.1 ± 1.2 -	11.41 ± 1.88 11.40 ± 1.75 12.50 ± 1.30 12.45 ± 1.20 - 10.02 ± 1.68
SLN-P-CA	-	10.00 ± 1.81

**Table 6:** Fluxes and diffusion coefficients of the indicated formulations.

Formulation	F ^1^ (µg/cm^2^/h)	D ^2^ (cm/h)
Sol-CA P-HA-CA P-CA SLN-CA SLN-P-CA	63.05 ± 4.41 40.23 ± 2.81 33.77 ± 2.36 26.21 ± 1.83 7.90 ± 0.55	126.11 ± 8.82 40.23 ± 2.81 33.77 ± 2.36 26.21 ± 1.83 7.90 ± 0.55

^1^ Flux; ^2^ Diffusion coefficient; Data are the mean of 6 independent Franz cell experiments.
